# Optimizing the Elastic Modulus Temperature Coefficient of Ti-45Nb Alloy by Aging-Induced Nano-Precipitation α Phase

**DOI:** 10.3390/ma17235871

**Published:** 2024-11-29

**Authors:** Fujian Guo, Guangyi Lu, Wenle Liu, Pan Zhang, Lei Jin, Chengjia Shang

**Affiliations:** 1 School of Materials Science and Engineering, Guangdong Ocean University, Yangjiang 529500, China; guofj@gdou.edu.cn; 2 Yang Jiang Advanced Alloys Laboratory, Yangjiang 529500, China; guangyi@yjlab.org.cn (G.L.); liuwl@yjlab.org.cn (W.L.); 3 State Key Laboratory for Advanced Metals and Materials, University of Science and Technology Beijing, Beijing 100083, China; 4 Shenzhen Fiyta Precision Technology Ltd., Shenzhen 518057, China; jinl@fiyta.com.cn

**Keywords:** TiNb alloy, heat treatment, phase transition, temperature coefficient of elastic modulus

## Abstract

Titanium–Niobium alloys have garnered extensive interest in various fields, such as aerospace, medical equipment, and scientific research instruments, due to their superior properties. Particularly, their anti-magnetic characteristics render them high potential in the watchmaking industry. The temperature coefficient of the elastic modulus of balance spring materials is a crucial parameter for assessing the impact of temperature on the properties of TiNb alloys. This study aims to explore the influence of heat treatment on the microstructural and elastic modulus temperature coefficient of the Ti-45Nb alloy. The results indicate that after short-term aging treatment, ω particles are enriched at grain boundaries and defects and are distributed in a necklace shape after erosion. After long-term aging treatment, the α phase appears in the material. The phase transformation process of low-temperature aging is β → β + β′ → β + ω → β + ω + α. The grain size of the material does not change significantly after different treatments. Additionally, the effect of heat treatment on material properties was studied by a low-temperature dynamic elastic modulus tester. The results showed that the temperature coefficient of the elastic modulus of the material in its original state was relatively high, ranging from 50~220 × 10^−6^·°C^−1^. After long-time aging treatment, the temperature coefficient of the elastic modulus of the material decreased significantly due to the appearance of the α phase. The temperature coefficient of the elastic modulus of the material after 48 h of heat preservation treatment fluctuated at 0 ± 30 × 10^−6^·°C^−1^. The internal control standard of excellent products in the industry is −11~35 × 10^−6^/°C. This study provides significant practical implications for the application of Ti-45Nb alloy in the watchmaking industry by adjusting the heat treatment temperature and time to study the effects on organizational evolution and the temperature coefficient of the elastic modulus.

## 1. Introduction

The hairspring of a watch can be regarded as the heart of a mechanical watch. It is a very thin spring that can control the balance wheel to move back and forth. In order to ensure the operation accuracy of the watch, the hairspring must have stable elastic properties, small elastic hysteresis, and a smaller temperature coefficient of elastic modulus [1]. In order to meet the requirements, the hairspring material is usually a constant elastic alloy [2]. For solid materials, the atomic thermal vibration will increase the atomic spacing, and the elastic modulus of the material will soften with the increase in temperature due to the lattice thermal vibration, which shows the well-known “hot soft, cold hard” characteristics [3]. The influence of temperature on the modulus is more significant. In addition to temperature, the material is also affected by the composition and microstructure, but the influence on the modulus is smaller than that of temperature. For application scenarios with large temperature changes, the modulus of materials will change significantly with temperature changes, which is very adverse to precision instruments and seriously affects their service behavior. At present, a variety of alloy materials can show a constant elastic effect, which is widely used in mechanical filters, tuning fork oscillators, clocks, hairsprings, and other fields [4,5]. In the early stage, the hairspring was mostly low-carbon steel, but it was gradually eliminated due to its shortcomings, such as being sensitive to magnetic force and temperature and easy to rust. In 1896, the French physicist Charles invented Ivar (64% Fe and 36% Ni), and then in 1950, the nivarox hairspring (cobalt nickel–chromium alloy) replaced Ivar. Up to now, with the progress of the watchmaking process, a variety of innovative materials are also emerging [6,7,8,9,10].

Titanium–Niobium alloy has attracted extensive attention in many fields such as aerospace, medical, and special elastic components because of its excellent properties such as shape memory effect, non-magnetic, corrosion resistance, low elastic modulus, nontoxicity, high fatigue limit, and good machinability [11,12,13,14,15,16,17,18,19,20]. At present, Switzerland has developed and applied Ti-Nb alloy to watch hairsprings and manufacture nivachron hairsprings. The elastic modulus of most metals and alloys will change with temperature. For the watch hairspring, the change in elastic modulus will affect the accuracy of travel time. Therefore, it is very important to control the temperature coefficient of the elastic modulus of materials. The microstructure, phase, and mechanical properties of binary TiNb alloy have been understood. According to the binary alloy phase diagram, TiNb alloys with all components are β-phase solid solutions with body-centered cubic structures above 882 °C. At a certain composition and temperature, the β phase can be transformed into a single α phase. At room temperature, TiNb alloy is a single ω phase or ω+β phase, which is related to Nb content. When the Nb content is high, a single β-phase solid solution can be obtained at room temperature. At present, there is no report on the effect of heat treatment on the temperature coefficient of elastic modulus of Ti-Nb alloy in the field of watches. This study studies the effect of heat treatment on the microstructure evolution and temperature coefficient of elastic modulus by adjusting the holding temperature and time, which has important practical significance for the application of Ti-Nb alloy.

## 2. Materials and Methods

The Ti-45Nb wire was formed by drawing, extrusion, and intermediate vacuum annealing. The Ti-45Nb alloy used in the experiment is a wire with a diameter of 5 mm, and the final state of the material is a hard material without heat treatment after stretching.

The microstructure observation samples with the size of 10 mm × 5 mm × 2 mm and the temperature coefficient test samples with the size of 5 mm in diameter and 80 mm in length were taken by wire cutting along the tensile direction of Ti-45Nb wire. According to the binary equilibrium phase diagram and metastable phase diagram of TiNb alloy [21,22,23], it can be seen from the phase diagram that TiNb alloys with different composition ratios have a single β phase solid solution with a body-centered cubic structure above 882 °C, and the β phase can be transformed into a single α hexagonal phase at a certain composition ratio and temperature, which is affected by the specific content of Nb. When the Nb content of the alloy is very high, it is a single β-phase solid solution, and the α phase is the main phase. Poor plasticity at the pinning center; the ω phase is a metastable phase and a pinning center, which can increase the hardness of the material. In the study of Lu [19], it was found that the Ti-rich hexagonal α phase precipitated from the heat treatment of TiNb alloy can reduce the temperature coefficient of elastic modulus, and the precipitation of α phase has a stronger impact on the temperature coefficient of elastic modulus than the reduction in dislocation density. A large number of defects, such as vacancies and dislocations, exist in the material without heat treatment after stretching, which accelerates the diffusion of elements, promotes the nucleation and growth of the α phase, and increases the non-uniform nucleation position to improve the uniformity of α phase distribution. According to the phase diagram, 420 °C is about the critical temperature of the α phase. Too high or too low temperature is unfavorable to the nucleation and growth of the precipitated phase; too short holding time leads to less precipitation of the α phase; and too long holding time leads to excessive growth of the first precipitated α phase. Combined with the research status at home and abroad, different heat treatment processes have been developed, as shown in Table 1.

Use a muffle furnace (boyuntong, kf1200, Nanjing, China) to heat treat the different samples in Table 1. Use sandpaper (400, 800, 1000, 1500, 2000 mesh) to polish the samples after heat treatment. Then, polishing spray with different particle sizes (2.5 μm, 0.5 μm) was used for polishing. The erosion solution with the ratio of HF:HNO_3_:H_2_O = 10%:30%:60% was used for surface erosion. A field emission scanning electron microscope (SEM, tescan, Mira3 LMH, Shanghai, China) equipped with an Oxford symmetry electron backscatter diffraction (EBSD) detector was used to observe the microstructure of the samples. The experimental parameters were as follows: Magnification 1000×, resolution 1024 × 1024, acceleration voltage 20 kV, working distance 16 mm, tilt angle 70°, step size 0.3 μm. EBSD data were analyzed and processed by channel-5 software (5.12.74.00) of the HKL company. The phase composition at room temperature was determined by X-ray diffraction (XRD, Shimadzu, 7000 s, Shanghai, China). The experimental parameters were as follows: Radiation source Cu-kα, voltage 40 kV, current 30 mA, angle 30°~90°, scanning speed 2°/min, step size 0.02°. A focused ion beam (FIB) is used for transmission sample preparation. Transmission electron microscopy (TEM, Thermo Fisher Scientific, jem-arm300f2, Waltham, MA, USA) was used to characterize the different phases more accurately. The experimental parameters were as follows: voltage 200 kV According to the temperature coefficient formula of elastic modulus (1) in the standard YB/T 5262-2011 [24] and the frequency temperature coefficient Formula (2) in GB/T 15006-2009 [25], f_1_ and f_0_ are measured by using a low-temperature dynamic elastic modulus tester (IET, Zhuosheng, 7015, Luoyang, China) and calculated to obtain the temperature coefficient of elastic modulus of the material.

(1) $$\beta =2\beta_{f}-8.5\times 10^{-6}/{}^{\circ}C$$

(2) $$\beta_{f}=(f_{1}-f_{0})/f_{0}(t_{1}-t_{0})$$

## 3. Results and Discussion

### 3.1. Microstructure

**Figure 1:**
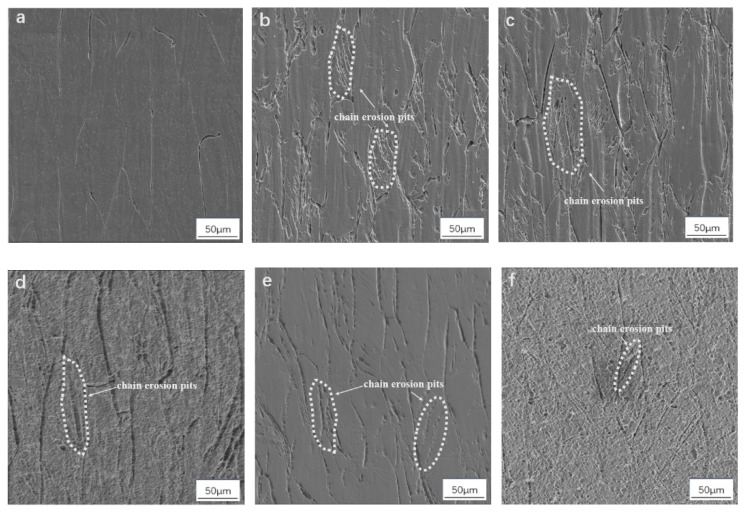
SEM photos of TiNb alloy with different heat treatment states. (**a**) Original state; (**b**) 400 °C insulation for 1 h; (**c**) 450 °C insulation for 1 h; (**d**) 420 °C insulation for 48 h; (**e**) 420 °C insulation for 60 h; (**f**) 420 °C insulation for 72 h.

Figure 1 shows the microstructure photos of TiNb alloy in its original state and after different heat treatments. It can be seen from the picture that the grains of Sample 1 original sample are elongated due to no heat treatment after stretching. Chain-like corrosion pits can be seen in Sample 2 and Sample 3 with the increase in holding temperature, and the higher the temperature, the more they appear. The proportion of chain-like corrosion pits in Sample 4, Sample 5, and Sample 6 with an increase in holding time does not increase significantly. Combined with Bin Tang’s research [26] on ω clusters of ti-45nb, it can be seen that sample 1 will produce a large number of lattice defects in the drawing process, such as vacancies and mismatched lattices, because vacancy clusters can serve as ω’s nucleation sites [27,28]. ω particles tend to enrich at grain boundaries and defects in subsequent heat treatment.

**Figure 2:**
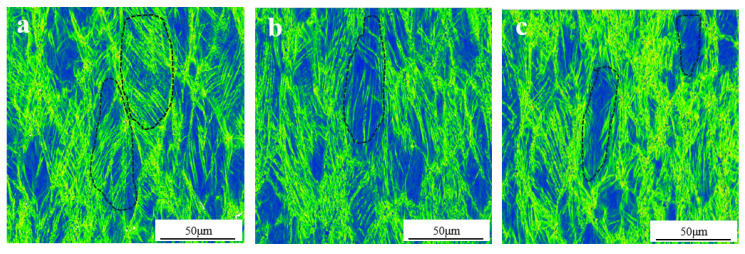

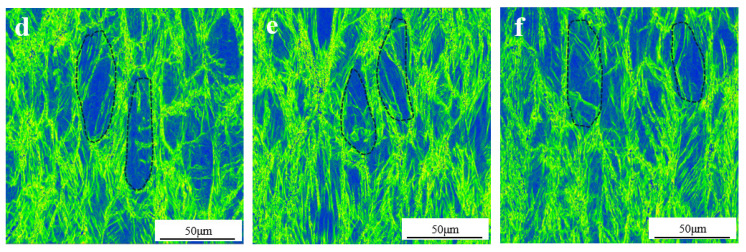
KAM photos of TiNb alloy with different heat treatment states. (**a**) Original state; (**b**) 400 °C insulation for 1 h; (**c**) 450 °C insulation for 1 h; (**d**) 420 °C insulation for 48 h; (**e**) 420 °C insulation for 60 h; (**f**) 420 °C insulation for 72 h.

KAM can qualitatively reflect the degree of homogenization in plastic deformation. The higher the value, the greater the degree of plastic deformation or the higher the defect density. Figure 2 shows the regions with high misorientation in the sample, which are shown in green and yellow. These regions produce tensile and compressive strains. There are a large number of feathery strain defects in the grains of the original sample, such as black dotted circles in Figure 2. As the holding time increases, the strain inside the grains decreases. The strain and defects in the material itself act as the nucleation sites of ω. During the heat treatment process, the ω phase appears at these positions and becomes the α phase with the increase in time.

### 3.2. Phase Characterization

**Figure 3:**
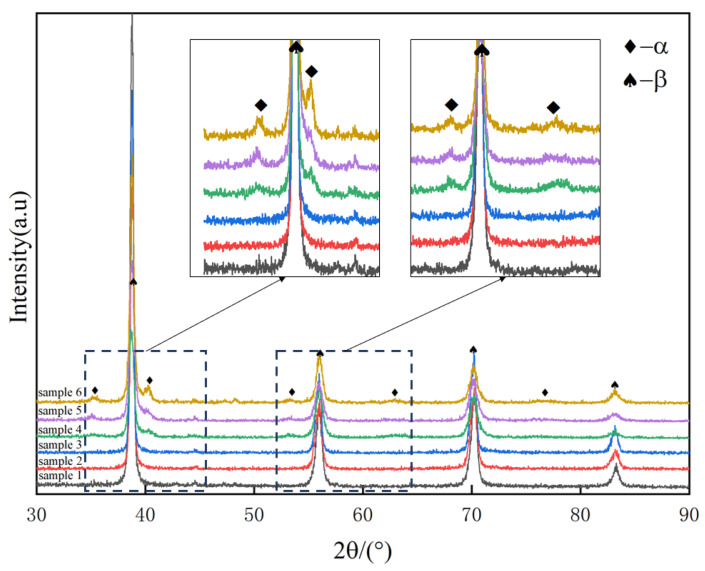
XRD photos of TiNb alloy with different heat treatment states.

In order to characterize the phase existing in the material, XRD was used to characterize the material. The characterization results are shown in Figure 3. Only a single β phase was found in the original state sample and in the sample with short-time insulation, and no ω phase was found, which is consistent with the study of Bin Tang [26]. After a long time of aging treatment, the α phase appeared in the material.

The β → α phase transformation process of the Nb-Ti alloy is relatively complex. The high Nb (β phase stable element) content TiNb alloy is a metastable β phase after rapid cooling from the β single-phase region. The enrichment and depletion of β stable elements first occurred in the matrix of metastable β phase during low-temperature aging, which differentiated into β phase and β‘ phase with the same crystal structure and different composition, maintaining the coherent relationship. During the continuous aging process, part of the displacement collapse of β‘ phase {111} interface depleted by β stable elements formed ω phase, followed by ω phase growth and coarsening, which was accompanied by the further collapse of β’ phase {111} interface and the diffusion of β stable elements between ω phase and matrix. ω phase is essentially a supersaturated solid solution of β stable elements in the α phase. With the continuous diffusion of β stable elements, the ω phase will eventually transform into a phase [29,30]. According to Figure 1, Figure 2 and Figure 3, the phase transformation process of low-temperature aging is β → β + β′ → β + ω → β + ω + α phase.

**Figure 4:**
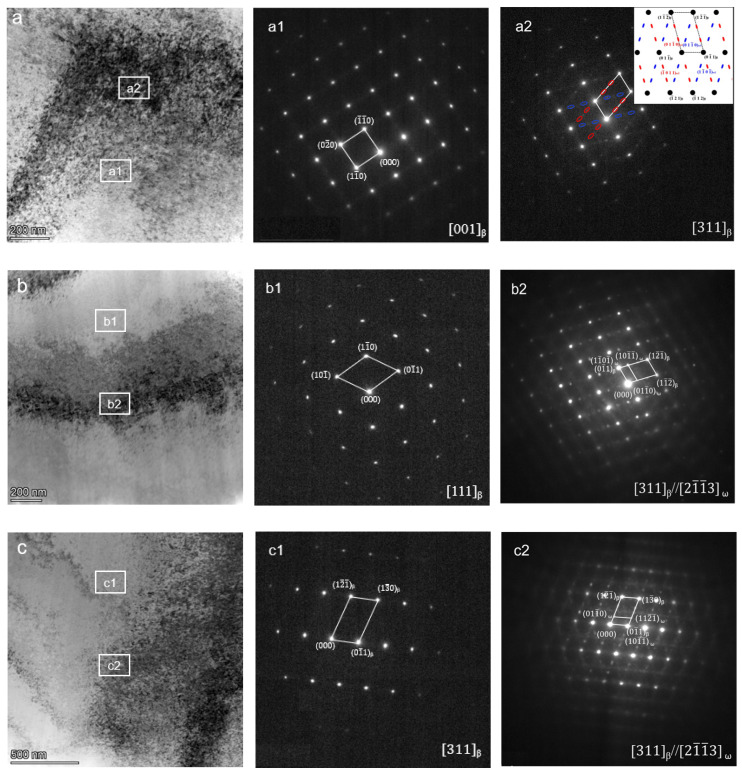
TEM images and diffraction patterns of TiNb alloy in different heat treatment states, (**a**) original state; (**b**) 400 °C insulation for 1 h; (**c**) 450 °C insulation for 1 h. (**a1**–**c1**) Matrix diffraction spot; (**a2**–**c2**) ω phase diffraction spot.

**Figure 5:**
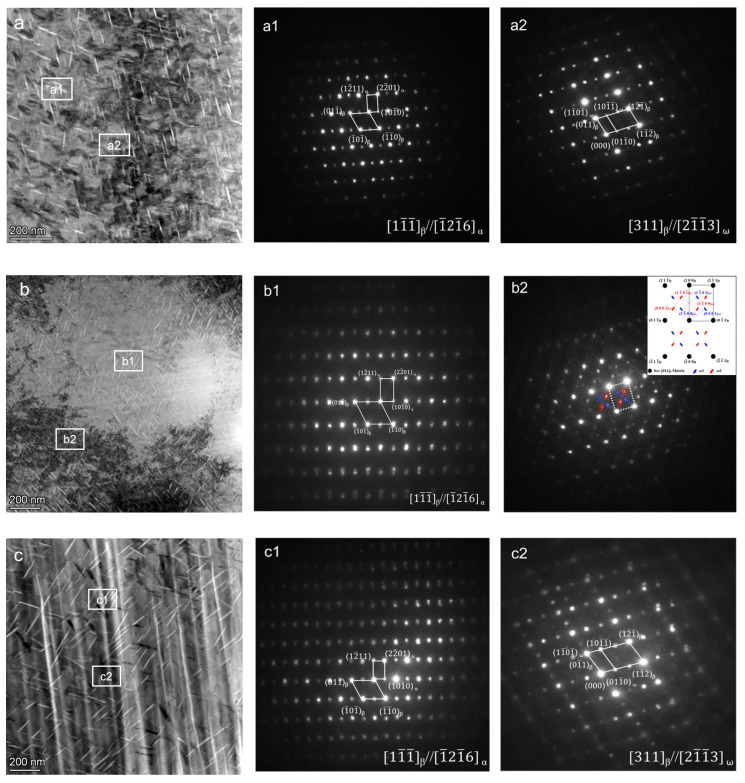
TEM images and diffraction patterns of TiNb alloy in different heat treatment states: (**a**) 420 °C insulation for 48 h; (**b**) 420 °C insulation for 60 h; (**c**) 420 °C insulation for 72 h. (**a1**–**c1**) α phase diffraction spot; (**a2**–**c2**) ω phase diffraction spot.

The phase composition inside the material was further accurately calibrated by transmission electron microscopy, and the results are shown in Figure 4 and Figure 5.

Figure 4 shows the microstructure and diffraction pattern of the original sample and the samples kept at 400 °C and 450 °C for 1 h. From the microstructure diagram, it can be seen that the microstructure of the three materials is basically the same. In the bright field image, it is shown as white and bright, clean areas and black areas. Combined with the calibration results of the diffraction pattern, it can be seen that in addition to the matrix being a single β phase, ω phase is also distributed. Because the ω phase is very fine and dispersed, the diffraction spots are weak and mixed with the matrix diffraction spots. No α phase was found in the three groups of samples.

Figure 5 shows the microstructure and diffraction pattern of the original sample after being kept at 450 °C for 1 h, 420 °C for 48 h, and 420 °C for 60 h. Compared with the microstructure of the three groups of samples in Figure 4, in Figure 5, the needle-like second phase appeared in the tissues of the three samples, and the black areas in the raw material were evenly distributed. By calibrating the diffraction pattern, it can be determined that the matrix is β phase, the acicular precipitate is α phase, and there are fine ω phases in the microstructure.

**Figure 6:**
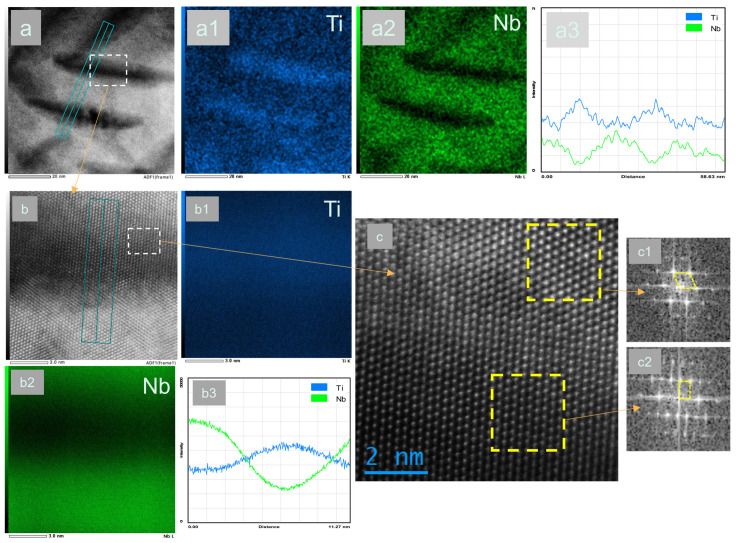
Energy spectrum and high-resolution photos of needle-like tissue of Sample d. (**a**,**a1**,**a2**,**a3**) High resolution map and surface and line distributions of Ti and Nb elements at low magnification; (**b**,**b1**,**b2**,**b3**) High resolution map and surface and line distributions of Ti and Nb elements at high magnification; (**c**,**c1**,**c2**) The high resolution image of the intersection of matrix and α and the inverse Fourier change image of two regions.

Figure 6 shows the structure energy spectrum and high-resolution photos of the original sample held at 420 °C for 48 h. From Figure 6a,b, it can be seen that the needle-like structure has high Ti content and low Nb content, and the α phase is rich in Ti and poor in Nb. It is determined again that the needle-like structure is precipitated in the α phase. The diffraction pattern obtained by inverse Fourier transform in the high-resolution image of Figure 6c also shows that the needle-like structure is α phase. The results show that the precipitates in Titanium alloy can play a strengthening role, the distribution of α phase is uniform and dispersed, and the crystal structure of α phase is hexagonal closed packed (HCP), which is different from the crystal structure of β phase in the matrix body-centered cubic (BCC), so the dislocation slip resistance will increase due to the dislocation of atoms on the slip surface. The section of the precipitated phase will produce a surface step with the width of the dislocation Burke vector, which will increase the interface area between the precipitated phase and the matrix, further increase the deformation resistance of the alloy, and enhance the strength of the alloy.

### 3.3. Material Properties

In mechanical watches, the travel time temperature coefficient C of the watch is required to be ≤1 s/°C·day, and its relationship with the temperature coefficient β of the elastic modulus of the material, the linear expansion coefficient A1, and the linear expansion coefficient A2 of the cycloidal material is shown in Equation (3), where A1 ≈ 8 × 10^−6^/°C, A2 ≈ 18 × 10^−6^/°C. C = 0 means that the travel time accuracy of the watch has nothing to do with the external temperature. Generally, Switzerland can achieve C within ±0.5, and the internal control standard of industrial standards and high-quality products is C within ±1. To make c = 0, β = 12 × 10^−6^/°C; If C is within ±0.5, β is (+0.5~+23.5) × 10^−6^/°C; If C is within ±1, β is (−11~+35) × 10^−6^/°C.

(3) $$c=86400(\frac{3a_{1}+\beta }{2}-a_{2})$$

**Figure 7:**
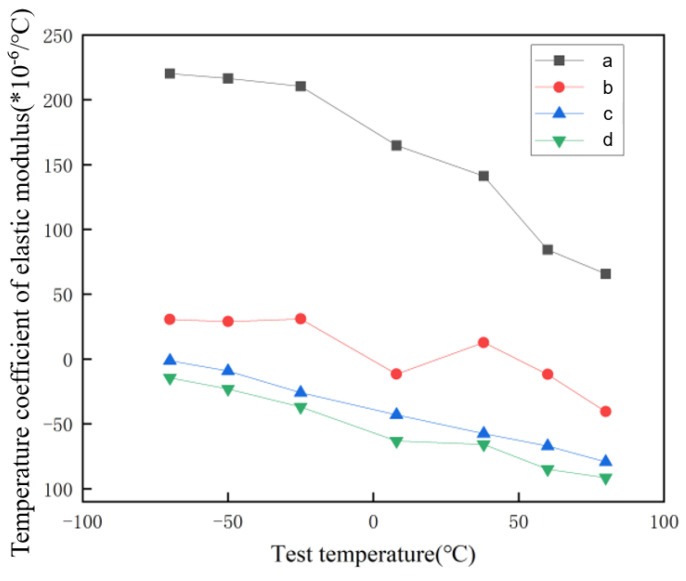
The distribution of elastic modulus temperature coefficient and test temperature of TiNb alloy in different states: (**a**) Original state; (**b**) 420 °C insulation for 48 h; (**c**) 420 °C insulation for 60 h; (**d**) 420 °C insulation for 72 h.

The alpha phase has a great impact on the performance of the material. The appearance of the alpha phase can reduce the temperature coefficient of the elastic modulus of the material. Figure 7 shows the distribution of the temperature coefficient of the elastic modulus of the material at different temperatures calculated from the IET measurement data. The temperature coefficient of the elastic modulus of the original sample at different temperatures is generally high (50~220 × 10^−6^/°C). Based on the influence of the temperature coefficient of the alpha relative elastic modulus, only Sample 1, Sample 4, Sample 5, and Sample 6 are tested.

Compared with the original material, the temperature coefficient of the elastic modulus of the material after aging treatment decreased significantly, which was consistent with the influence of the temperature coefficient of α relative elastic modulus. With the increase in aging time, the temperature coefficient of the elastic modulus of the material tested at different temperatures decreased. The temperature coefficient of the elastic modulus of the material kept at 420 °C for 48 h fluctuated at 0 ± 30 × 10^−6^/°C, indicating that the elastic modulus of the material was little affected by temperature.

## 4. Conclusions

(1) The as-received Ti-45Nb alloy, after short-term heat treatment, forms necklace-like corrosion pits within its microstructure, which are identified as ω phase clusters predominantly enriched near the grain boundary. Furthermore, after long-term aging treatment, the ω phase persists, and the formation of a new α phase is observed within the alloy, which significantly enhances the material’s hardness.
(2) The elastic modulus temperature coefficient of the as-received Ti-45Nb alloy is relatively high (50~220 × 10^−6^/°C). After long-term aging treatment, the presence of the α phase significantly reduces the alloy’s elastic modulus temperature coefficient (−80~50 × 10^−6^/°C). Notably, the material treated for 48 h exhibits an elastic modulus temperature coefficient fluctuating between -30 and 30 × 10^−6^/°C, indicating minimal sensitivity to temperature changes, which is suitable for the fabrication of watch balance springs.

## Figures and Tables

**Table 1:** Ti-45Nb heat treatment parameters.

Samples	Temperature/°C	Time/h
1	Not handled	Not handled
2	400	1
3	450	1
4	420	48
5	420	60
6	420	72

## Data Availability

The original contributions presented in this study are included in the article. Further inquiries can be directed to the corresponding author.

## References

[1] Zhao J.H., Zhong F., Wu Z.P., Jiang T. (2019). Development status and trend of hairspring materials. Sci. Technol. Innov..

[2] The Constant Elastic Alloy Group of the Instrument Materials Research Institute of the First Machine Department (1971). Introduction to Constant Elastic Alloys. Instrum. Mater..

[3] Kittel C., Mceuen P. (2018). Introduction to Solid State Physics.

[4] Roy R. (2003). Very Low Thermal Expansion Coefficient Materials. Annu. Rev. Mater. Res..

[5] Nakamura Y. (1975). The invar problem. IEEE Trans. Magn..

[6] Xu J.Z. (1988). New Progress in Constant Elastic Alloys. Shanghai Met. (Steel Div.).

[7] Li Z.Z., Yu Y.G., Sun B.H. (1985). Research on High Elasticity and High Antimagnetic Windlass Materials. Instrum. Mater..

[8] Feng Y.L. (1987). Constant elastic alloy for foreign watch hairspring. Shanghai Gangyan.

[9] Zhong X.Y. (1989). Research on Elastic Alloy RC-1 for High Stability Anti magnetic Watch Windings. Instrum. Mater..

[10] He Q.H., Li F., Cai X.N., Zhang S.Q., Wang H., Zhao Z., Dong M.L., Zhao A.Z. (2020). Effect of Time Effective Treatment on TBe_ The influence of microstructure and properties of 2-micron hairspring materials. J. Mater. Heat Treat..

[11] Wilson M.N. (2008). NbTi superconductors with low ac loss: A review. Cryogenics.

[12] Santra S., Davies T., Matthews G., Liu J., Grovenor C.R.M., Speller S.C. (2019). The effect of the size of Nb Ti filaments oninterfacial reactions and the properties of In Sn-based superconducting solder joints. Mater. Des..

[13] Nannini M., Cloez H., Girard S., Roux C., Serries J.-P., Tena M., Zani L., Mossang E. (2009). Characterization of industrial NbTi strands at variable field for JT-60SA toroidal field coils. Fusion Eng. Des..

[14] Zhou F., Cheng J., Dai Y., Wang Q., Yan L. (2014). Numerical simulation of mold shape’s influence on NbTi cold-pressing superconducting joint. Phys. C Supercond. Its Appl..

[15] Zhang Z.Y., Matsumoto S., Choi S., Teranishi R., Kiyoshi T. (2011). A new structure for a magnetic field concentrator using NbTi sheet superconductors. Phys. C Supercond..

[16] Huang T.W., Zhou F.Y., Zhao L.Z. (1982). Application of elastic niobium alloy on corrosion resistance of the instrument. J. Instrum. Mater..

[17] Huang T.W. (1985). Constitution of elastie Nb alloy Nb-40Ti5.5A1. Acta Metall. Sin..

[18] Wang S.L. (1990). Development and Application of Constant Elastic Alloy.

[19] Lv Q.F. (1980). Development Overview of Niobium Based Elastic Alloys Abroad.

[20] Li J.F., Zhang P.X., Liu X.H., Li J.S., Feng Y., Wang T.C., Du S.J., Liu W.T. (2009). Research progress on NbTi superconductors for magnets. Mater. Rev..

[21] Bönisch M., Calin M., Waitz T., Panigrahi A., Zehetbauer M., Gebert A., Skrotzki W., Eckert J. (2016). Thermal stability and phase transformations of martensitic Ti–Nb alloys. Sci. Technol. Adv. Mater..

[22] Bahador A., Hamzah E., Kondoh K., Bakar T.A.A., Yusof F., Imai H., Saud S.N., Ibrahim M.K. (2017). Effect of deformation on the microstructure, transformation temperature and superelasticity of Ti–23 at% Nb shape-memory alloys. Mater. Des..

[23] Bönisch M., Panigrahi A., Calin M., Waitz T., Zehetbauer M., Skrotzki W., Eckert J. (2017). Thermal stability and latent heat of Nb–rich martensitic Ti-Nb alloys. J. Alloys Compd..

[24] (2012). Constant Elastic Alloy 3J53Y Wire for Watch Hair.

[25] (2009). General Provisions for Dimensions, Shape, Surface Quality, Test Methods, and Inspection Rules of Elastic Alloys.

[26] Tang B., Kou H.C., Zhu Z.S., Li J.S. (2011). Enrichment behavior of ω clusters in cold-drawn Ti-45Nb filaments. Mater. Sci. Forum.

[27] Sukedai E., Yoshimitsu D., Matsumoto H., Hashimoto H., Kiritani M. (2003). β to ω phase transformation due to aging in a Ti–Mo alloy deformed in impact compression. Mater. Sci. Eng. A.

[28] Prima F., Debuigne J., Boliveau M., Ansel D. (2000). Control of omega phase volume fraction precipitated in a beta titanium alloy: Development of an experimental method. J. Mater. Sci. Lett..

[29] Fan Z., Miodownik A.P. (1994). TEM study of metastable β-phase decomposition in rapidly solidified Ti-6Al-4V alloy. J. Mater. Sci..

[30] Devaraj A., Nag S., Srinivasan R., Williams R.E.A., Banerjee S., Banerjee R., Fraser H.L. (2012). Experimental evidence of concurrent compositional and structural instabilities leading to ω precipitation in titanium–molybdenum alloys. Acta Mater..

